# A General Model of Codon Bias Due to GC Mutational Bias

**DOI:** 10.1371/journal.pone.0013431

**Published:** 2010-10-27

**Authors:** Gareth A. Palidwor, Theodore J. Perkins, Xuhua Xia

**Affiliations:** 1 Ottawa Hospital Research Institute, Ottawa, Canada; 2 Department of Biology and Center for Advanced Research in Environmental Genomics (CAREG), University of Ottawa, Ottawa, Canada; University of Nottingham, United Kingdom

## Abstract

**Background:**

In spite of extensive research on the effect of mutation and selection on codon usage, a general model of codon usage bias due to mutational bias has been lacking. Because most amino acids allow synonymous GC content changing substitutions in the third codon position, the overall GC bias of a genome or genomic region is highly correlated with GC3, a measure of third position GC content. For individual amino acids as well, G/C ending codons usage generally increases with increasing GC bias and decreases with increasing AT bias. Arginine and leucine, amino acids that allow GC-changing synonymous substitutions in the first and third codon positions, have codons which may be expected to show different usage patterns.

**Principal Findings:**

In analyzing codon usage bias in hundreds of prokaryotic and plant genomes and in human genes, we find that two G-ending codons, AGG (arginine) and TTG (leucine), unlike all other G/C-ending codons, show overall usage that decreases with increasing GC bias, contrary to the usual expectation that G/C-ending codon usage should increase with increasing genomic GC bias. Moreover, the usage of some codons appears nonlinear, even nonmonotone, as a function of GC bias. To explain these observations, we propose a continuous-time Markov chain model of GC-biased synonymous substitution. This model correctly predicts the qualitative usage patterns of all codons, including nonlinear codon usage in isoleucine, arginine and leucine. The model accounts for 72%, 64% and 52% of the observed variability of codon usage in prokaryotes, plants and human respectively. When codons are grouped based on common GC content, 87%, 80% and 68% of the variation in usage is explained for prokaryotes, plants and human respectively.

**Conclusions:**

The model clarifies the sometimes-counterintuitive effects that GC mutational bias can have on codon usage, quantifies the influence of GC mutational bias and provides a natural null model relative to which other influences on codon bias may be measured.

## Introduction

Codon bias, the unequal usage of synonymous codons, varies widely between species and, in some cases, between different regions of a genome in a single species [1]. Factors influencing codon bias include selection for translational accuracy and efficiency [2]–[6] and GC bias, on a genomic level in prokaryotes [7] and on a regional or isochoric level in vertebrates [8].

The influence of GC bias is a major influence on codon bias both in human [9] and prokaryotic genomes [7], resulting in a close association between GC% at the third codon position, also called GC3 [10] and GC bias (genomic GC% in prokaryotes or isochoric GC% in mammals). As all amino acids (with the exception methionine and tryptophan) allow GC-changing synonymous substitutions in the third position, this has led to a common belief that the use of synonymous G/C-ending codons should increase in frequency with increasing GC bias, while usage of A/T-ending codons should decrease [11]. Though this is a reasonable assumption for the codons of most amino acids, those that allow GC-changing synonymous substitutions in the first and third codon positions, arginine and leucine, may be expected to act differently. In particular, those codons which have one A or T and one G or C in the first and third synonymous positions will have a conflicted response to GC bias; this effect has not previously been modeled.

The recent rapid growth in the availability of both partial and full genomic sequences has allowed for broad studies of codon usage subject to GC bias across large numbers of species. Hershberg & Petrov [12] identified favored codons in prokaryotes and fungi and showed that they are strongly related to the species' intergenic GC content. Knight et al. [13] modeled codon and amino acid usage as a function of GC mutational bias in bacteria, prokaryotes and eukaryotes showing that GC content drives codon and amino acid usage and provide a model of usage by compositional class, but did not directly address codon bias.

In this paper we generate a continuous-time Markov chain model of codon bias as a function of imposed GC bias for all amino acids. We test the model by comparing it with codon bias for prokaryote and plant genomes and the genes of the human genome. Finally we discuss possible causes for observed variations from the model.

## Results

### General patterns of codon usage

**Figure 1:**
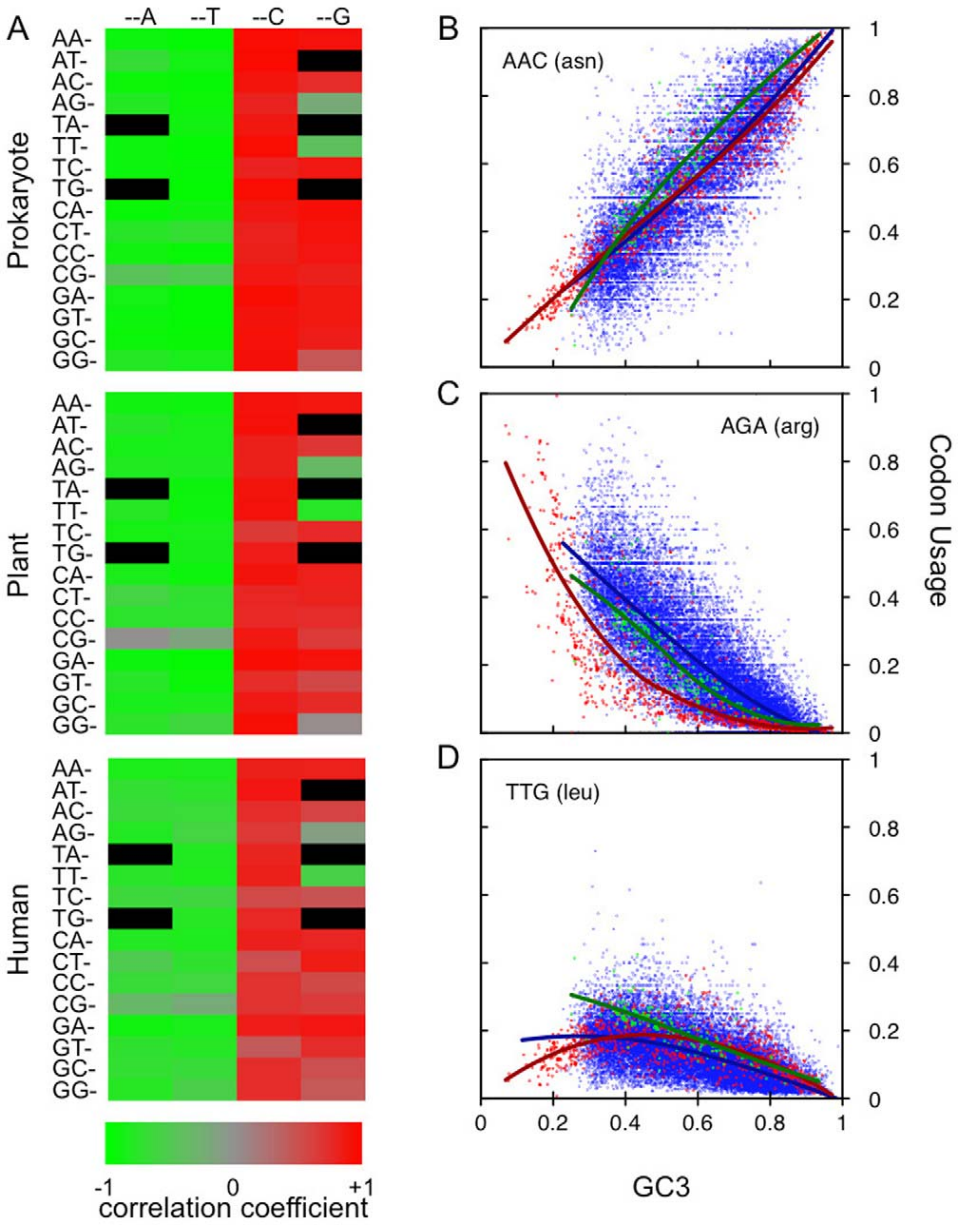
Relationships between codon usage and GC3. (A) Heat maps of correlation values for codon usage vs GC3 for bacteria and plant genomes and human genes. The color and intensity indicates type and degree of correlation: red indicates positive, green negative. Black fields are stop and non-degenerate codons (tryptophane and methionine). AGG and TTG are the only G/C-ending codons having negative correlation with GC3. (B) Codon usage frequency for the asparagine codon AAC (linear). Red, green and blue points and lines are, respectively, bacterial, plant and human scatter plots and LOESS fits of codon usage frequency versus GC3. This is also the case for Figures 1C and 1D. (C) Codon usage frequency for the arginine codon AGA (nonlinear, but monotone). (D) Codon usage frequency for the leucine codon TTG (nonlinear and non-monotone).

Figure 1A displays heatmaps of the correlation coefficients between codon usage and GC bias (across species for prokaryotes and plants, and across genes for human). In all three analyses, the usage of nearly all G- and C-ending codons is strongly positively correlated with GC bias, and conversely for A- and T-ending codons. However, two G-ending codons, AGG (arginine) and TTG (leucine), defy this overall trend consistently across the three different groups of data by showing a negative correlation between usage and GC bias. Two other arginine codons, CGA and CGT, display the expected negative correlation, but the correlation is very weak.

Looking at the usages of individual codons in more detail, we find additional mysteries. For many codons, usage is well modeled as a linear function of GC bias. For example, Figure 1B shows a scatter plot of the usages of the AAC asparagine codon versus GC bias for prokaryote and plant genomes as well as human genes. However, other codons show distinctly nonlinear usage profiles. For example, the arginine codon AGA shows a nonlinear upwardly curving usage as a function of GC bias (Figure 1C) particularly among prokaryotes. The leucine codon TTG, shows a non-monotone usage pattern in this case, with peak usage occurring in genomes with nearly neutral GC bias (Figure 1D).

**Figure 2:**
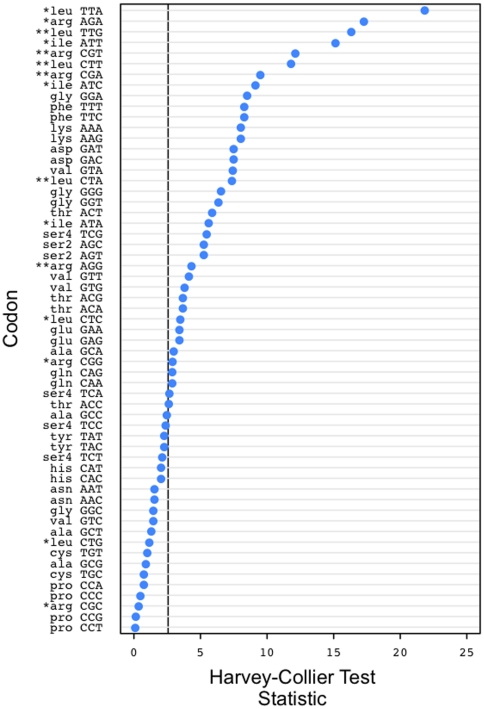
Graph of Harvey-Collier test statistic for codon bias as a function of GC3 for prokaryotes, ordered by decreasing magnitude. Low values indicate linearity, high values indicate nonlinearity. * and ** indicate codons that our model predicts to be nonlinear and nonlinear, non-monotone respectively.

**Figure 3:**
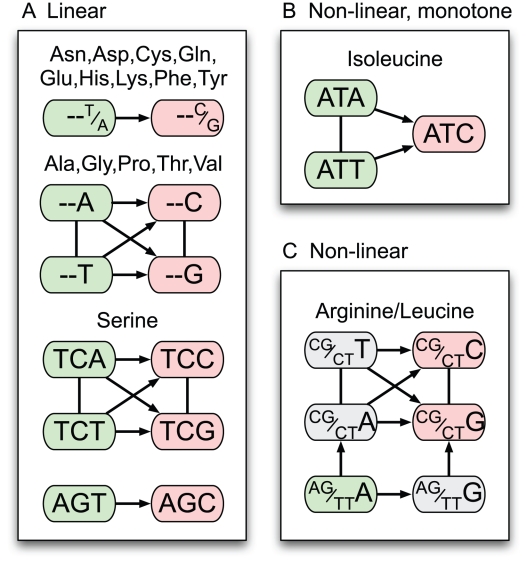
Networks of synonymous single site substitutions for all amino acids. GC increasing substitutions are indicated with an arrow. GC preserving substitutions are represented by a line. For amino acids with 2 and 4 codons as well as serine, G/C-ending codons are pink, and A/T-ending codons green. For arginine and leucine, codons with G/C in both synonymous positions (2×GC codons) are red, those with only one G/C in a synonymous position (1×GC) are grey, and those with A/T in both synonymous positions (0×GC) are green. (A) Amino acids whose codons are predicted to have a linear response to GC mutational bias. (B) Isoleucine, whose codons are predicted to have a nonlinear but monotone response to GC mutational bias. (C) Arginine and leucine, whose codons are predicted to have nonlinear, and in some cases non-monotone, responses to GC mutational bias.

We used the Harvey-Collier test to assess the null hypothesis of linear usage for all codons (see Figure 2). The test shows that a large number of codons exhibit some degree of nonlinear usage in prokaryotes as a function of GC bias, though this may be influenced by violation of the constant-variance assumption made by the test or by the large number of data points involved. However, a number of codons, particularly belonging to leucine, isoleucine and arginine, show very strong deviations from linearity. We hypothesized that the unusual responses of these isoleucine, arginine and leucine codons may result from the structure of possible synonymous single site substitutions within these amino acids (Figure 3). Notably, arginine and leucine are the only amino acids that allow GC-changing synonymous substitutions in the first as well as the third position (Figure 3C). Isoleucine is the only amino acid with three codons and unequal numbers of G/C- and A/T-ending codons (Figure 3B). The remainder of the amino acids have equal numbers of A/T and G/C-ending codons and only allow GC-changing synonymous substitutions in the 3rd codon position. The usage of these codons is in many cases linear, or at least much closer to linear than shown by many of the leucine, isoleucine and arginine codons.

### A Model for Codon Usage Based on Synonymous Mutations

We propose a continuous-time Markov chain model of codon evolution under point mutations that explains the observations above. We assume that no non-synonymous mutations are allowed, so that all variation in usage is due to synonymous mutations. Though there are mechanisms by which synonymous mutation may affect protein function, they are more likely to be effectively neutral than non-synonymous mutations [14]. Using this simplifying assumption, we are able to explain the major patterns of codons used without recourse to the added complexity of including non-synonymous mutations.

Consider the codons relating to any particular amino acid. For any two codons 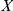 and 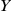 that differ by a single nucleotide, we assume that the rates of mutation from 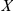 to 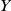 and from 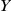 to 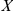 are the same if both codons have the same total number of Gs and Cs 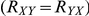. If, however, Y has one more G or C than 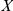, then we assume that mutation from 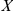 to 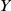 happens at 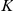 times the rate of mutation from 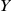 to 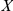 (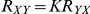). Here, 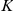 is related to the GC bias 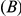 as 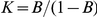. 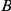 ranges from total A/T bias to total G/C bias 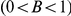. Finally, we assume that the usages of the codons of each amino acid, in a set of genes or a genome, are equal to the equilibrium frequencies of those codons under the model. Letting 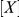 and 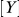 denote the usages of codons 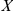 and 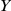, then 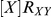 = 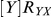.

From the continuous-time Markov chain models for every amino acid except serine, one can solve for the equilibrium usage of all codons, as a function of GC bias 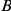, based on the set of possible synonymous single site mutations for each amino acid (Figure 3). The equilibrium solutions are summarized in Table 1. Importantly, the predicted equilibrium usages of every codon depend only on the GC bias. They are not affected, for example, by differering transition and transversion mutation rates; as long as the rates are non-zero the equilibrium solutions are the same. Nor are there any free parameters of the model that need to be determined from the data, except, of course, for the GC bias itself.

**Table 1:** Equilibrium solutions for codon frequencies.

Amino acid/class	Codon	Frequency
Two-codon	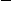 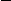 A or 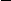 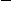 T	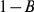
	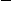 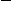 C or 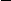 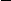 G	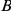
Four-codon	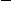 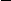 A and 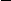 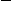 T	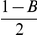
	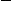 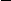 C and 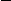 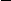 G	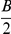
Isoleucine	ATA and ATT	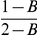
	ATC	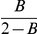
Arginine/Leucine	AGA and TTA	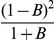
	CGA, CGT, AGG,	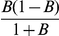
	CTA, CTT and TTG	
	CGG, CGC,	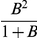
	CTG and CTC	

Serine raises a small problem for our model in that it is the only amino acid that consists of disconnected blocks of codons—one set of four codons and another of two codons—which cannot be reached from each other by any combination of synonymous point mutations. As a consequence, the model can only explain the usages of the TCA, TCC, TCG and TCT codons with respect to each other, and of the AGC and AGT codons with respect to each other. The relative usages of the first four compared to the last two is beyond the scope of our model. For the remainder of this article, we will treat the four TC-beginning codons as if they belong to a single amino acid, which we call serine4, and the two AG-beginning codons as if they belong to a different amino acid, which we denote serine2. Implicitly, this means that we redefine the usage of each TC-beginning codon as its number of occurrences by the total number of occurrences of TC-beginning codons, and likewise for the AG-beginning codons.

### Linear Usage Predicted for all 2-Codon and 4-Codon Amino Acids

For all the two-codon and four-codon amino acids, including the serine2 and serine4 groups, the model predicts codon usage that is linearly increasing or decreasing in GC bias, B, depending on whether the codon ends with an A/T or with a G/C. Consider first the two-codon amino acids. Each has one A/T-ending codon, which we will denote by 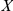, and one G/C-ending codon, which we will denote by 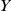. Because 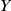 has one more G/C than 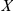, the model asserts that the mutation rates satisfy 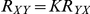. At equilibrium, the flux from 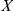 to 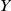 must match the flux from 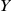 to 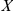:
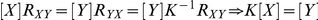
The equilibrium frequencies must also sum to one, so we have:
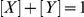
Combining these two equalities, and recalling that 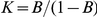 we can solve for the frequencies of 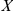 and 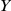:
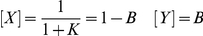
As one would expect, usage of the A/T-ending codon decreases linearly with increasing GC bias, with 0% usage in completely GC-biased situations (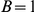) and 100% usage in completely AT-biased situations (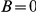). The opposite holds for the G/C-ending codon.

For a four-codon amino acid, the derivation is similar. The model's assertion about mutation rates implies six different equalities, corresponding to the six possible pairs among the four codons. Let us focus on three:
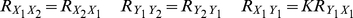
The remaining three relationships turn out to be redundant with these three, as the reader can easily verify. At equilibrium, the balance of fluxes imply that:
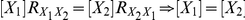


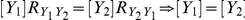


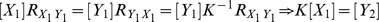
Combining these with the fact that the frequencies sum to one, 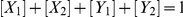, we can solve to obtain:
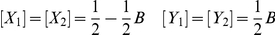
The usages of the A/T-ending codons are predicted to be exactly half of the usage of a single A/T-ending codon in a two-codon amino acid, and likewise for the G/C-ending codons.

**Figure 4:**
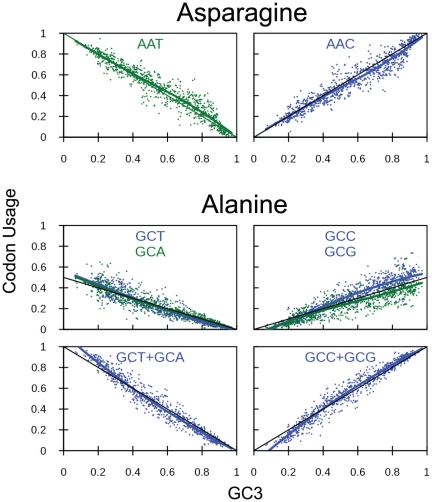
Codon usage frequency for asparagine and alanine based on prokaryotic data. The x-axis is genomic GC3 as an estimate of GC bias, The y-axis is usage frequency. The colored points and lines correspond to the observed codon usage frequencies for a given GC3 and the corresponding loess fit respectively. The black line is the model prediction. (A) Asparagine AAT codon. (B) Asparagine AAC codon. (C) Alanine GCT and GCA codons. (D) Alanine GCC and GCG codons. (E) The sum of the GCT + GCA alanine codons. (F) The sum of the GCC + GCG codons.

Figure 4 shows example codon usages for asparagine, a two-codon amino acid, and alanine, a four-codon amino acid in the prokaryotic data. For asparagine, the model matches the observed usages with great accuracy. For alanine the overall trends of the A/T-ending and G/C-ending codons are well-captured, including the fact that their usages are approximately linear and have half the slope of the two-codon usages. However, one also observes that in the most GC-biased situations (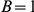), the two G/C-ending codons do not received precisely one half of the usage. In some species, the G-ending codon receives more of the usage, while in other species the C-ending codon receives more usage. This inequality in usage between the two G/C-ending codons is correlated to their overall usage. A similar phenomenon occurs with the A/T-ending codons. Interestingly, for a four-codon amino acid, if one sums the usages of the two A/T-ending codons and of the two G/C-ending codons, one obtains usages that very accurately match what one would expect for a two-codon amino acid.

### Nonlinear, but Monotone, Usage Predicted for Isoleucine Codons

**Figure 5:**
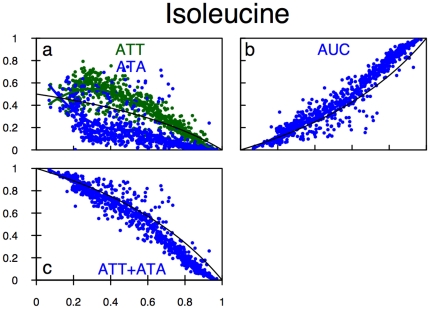
Codon usage frequency for isoleucine based on prokaryotic data. The x-axis is genomic GC3 as an estimate of GC bias, The y-axis is usage frequency. The colored points and lines correspond to the observed codon usage frequencies for a given GC3 and the corresponding loess fit respectively. The black line is the model prediction. (A) ATT and ATA codons. (B) ATC codon. (C) The sum of the ATA+ATT codon usages.

For isoleucine, the derivation is similar, with the notable difference being the unequal numbers of G/C- and A/T-ending codons. The synonymous mutation relationships are shown in Figure 3B. Let 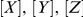 represent the equilibrium frequencies for the codons ATA, ATT and ATC respectively. Our assumptions regarding mutation rates imply that 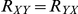 and 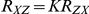. The flux balance at equilibrium then implies:
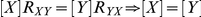


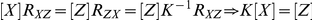
Using 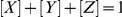 and 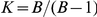, we obtain
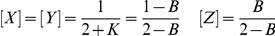
The usage curves are shown in Figure 5, along with the prokaryote data. The predicted and observed usages of ATC match very well showing positive slope and mild upward curvature. For ATA and ATT, the observed usages are decreasing in GC bias 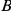, particularly for 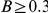. For that range of 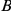, the usage curves also show the downward curvature predicted by the model, particularly ATT. However, the usage of ATT is approximately double that of ATA, in contradiction to the model prediction. In the vicinity of 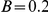, there is a sudden “correction” of the relative usages, so that for the most A/T-biased species for which we have data, the usages of both ATT and ATA are approximately as predicted by the model. Despite these complicated patterns in ATA and ATT usage, as shown in Figure 5C, the summed usages of ATA and ATT match very well the model's prediction for their sum, over the whole range of GC bias and is notably nonlinear.

### Nonlinear, and in Some Cases Nonmonotone, Usage Predicted for Arginine and Leucine Codons

Arginine and leucine are notable as the only amino acids which allow synonymous, GC-changing point mutations in both the first and third codon positions as shown in Figure 3C.

Let 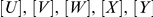 and 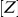 represent the equilibrium frequencies of the arginine codons AGA, CGA, CGT, AGG, CGG and CGC respectively or, equivalently, the equilibrium frequencies of the leucine codons TTA, CTA, CTT, TTG, CTG and CTC respectively. Our assumptions regarding mutation rates imply nine different equalities, corresponding to the nine possible pairs among the nine codons. Only four non-redundant equalities must be specified: 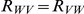, 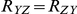, 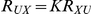 and 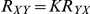. The flux balance at equilibrium then implies:
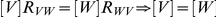


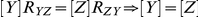


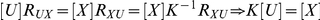


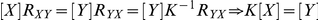
Substituting the above into 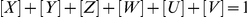 and solving for 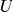 gives:
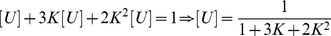
Substituting 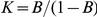 and solving for codon frequencies gives:
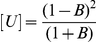


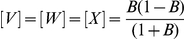


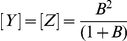
For both arginine and leucine, three distinct usage patterns are predicted, depending on whether a codon has total of zero, one or two Gs and Cs in the first and third positions, those that can vary synonymously. We will refer to these codon classes as 0×GC, 1×GC and 2×GC respectively, corresponding to the three solutions above.

**Figure 6:**
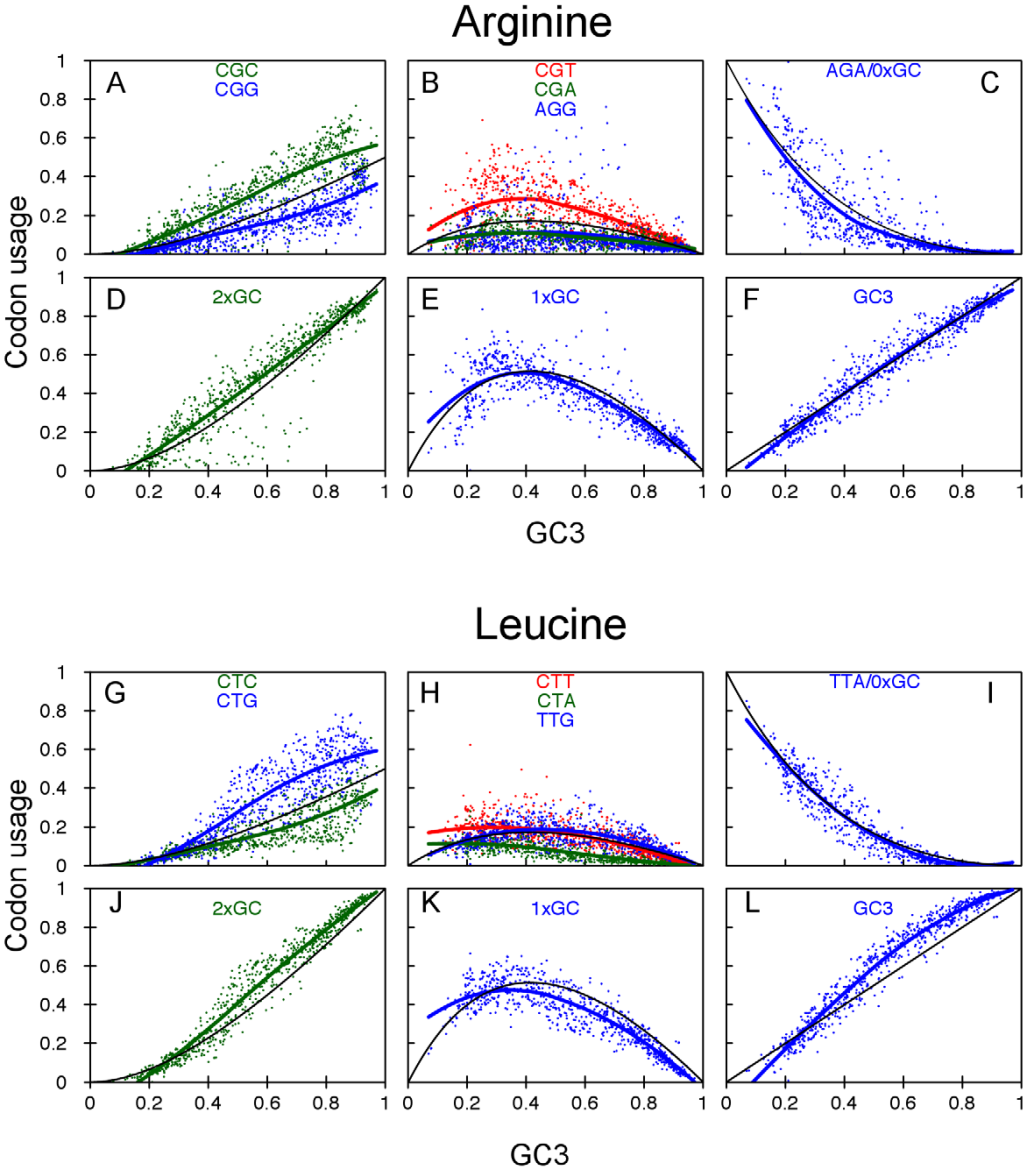
Codon usage frequency for arginine and leucine based on prokaryotic data. The x-axis is genomic GC3 as an estimate of GC bias, the y-axis is usage frequency. The colored points and lines correspond to the observed codon usage frequencies for a given GC3 and the corresponding loess fit respectively. The black line is the model prediction. (A) 2×GC arginine codons (those with two G/C in the 1st or 3rd codon position). (B) 1×GC arginine codons (those with one G/C in the 1st or 3rd codon position). (C) The 0×GC arginine codon (with no G/C in the 1st or 3rd codon position). (D) The sum of 2×GC arginine codons. (E) The sum of 1×GC arginine codons. (F) GC3 for arginine codons. (G) 2×GC leucine. (H) 1×GC leucine codons. (I) 0×GC leucine. (J) The sum of 2×GC leucine codons. (K) The sum of 1×GC leucine codons. (L) GC3 for leucine codons.

0×GC (arginine: Figure 6C, leucine: Figure 6I) and 2×GC (arginine: Figure 6A, leucine: Figure 6G) usage curves are monotonic and nonlinear decreasing and increasing respectively with increasing GC3 described by 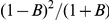 and 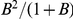 respectively. 1×GC codons (arginine: Figure 6B, leucine: Figure 6H) show a non-monotone, asymmetric, concave distribution described by 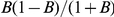 with a peak at 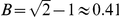, below neutral bias 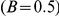.

Intuitively, the 1×GC codons show peaked usage because when GC content is low the 0×GC codons are strongly favored, and when GC content is high the 2×GC codons are strongly favored. The asymmetry of the peak position is due to the fact that there are two 2×GC codons in each of arginine and leucine but only one 0×GC codon. The peak position is notable as a correlation of such a curve over the range of 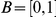 will be negative, as shown for AGG (Figure 6B) and TTG (Figure 6H). The negative correlation is stronger when the data is richer in GC sequences, as is the case with the human data. This result provides an alternative interpretation of the observations of Kliman & Bernal [11] who reported that AGG and TTG codons in human are negatively correlated with GC3, intronic GC and expression and postulated that this may be the result of selection on these codons. AGG and TTG are the two G-ending 1×GC codons; their observed usage frequency patterns are predicted by our model for human as well as prokaryote and plant genomes.

## Discussion

Motivated by some unexpected observation on codon usage, we have developed a codon usage model based on GC-biased synonymous point mutations. The model makes a number of predictions. The most important and notable prediction is that two G-ending codons (AGG, TTG) will show decreasing usage with increasing GC bias as indicated by GC3. The second prediction of our model is that some codon frequencies will be nonlinear and others linear as a function of GC bias. The third prediction of our model is that the per-amino acid GC3 should be linear for all amino acids with the exception of isoleucine.

Our model predictions are most accurate in the prokaryotic data due to the large number of species in the data set, the fact that each species' data point has a high codon counts (at least 50 coding sequences and usually much more) and they cover a broad range of GC3 values. The plant data is much more limited, consisting of fewer genomes covering a narrower range of GC3 values. The human data as well has a narrower GC3 values than the prokaryotic data. Though the human data consists of many more data points, each data point is based on the relatively small number of codons in a human gene. As a result of this and the ratios used for codon frequency calculations, there are strong stochastic effects and the data is quite noisy resulting in lower prediction accuracy. Despite the limitations of the data, the key prediction of our model that AGG and TTG should have negative correlation with respect to GC3 holds true for all the data sets as clearly shown for prokaryotic, plant and human data in Figure 1A.

Figure 2, which shows the results of the Harvey-Collier test for linearity, shows that the 8 most nonlinear codons as a function of GC3 are in the list of 12 that our model predicts to be nonlinear. While the results for plant and human data are less definitive (see Table S1 for data and Figures S4, S5 and S6 for human, plant and prokaryote data respectively), we attribute this to the properties of those data sets, as described above.

For all of the model solutions except isoleucine, the usages of G/C-ending codons sum to 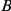, whereas the usages of A/T-ending codons sum to 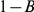. Thus, the observed overall GC bias in the third codon position is predicted to be followed by each amino acid individually (see Figures S1, S2 and S3 for human, plant and prokaryote per-amino acid GC3 vs GC3 graphs respectively). Indeed, this is true for arginine and leucine as well, though somewhat surprising due to the nonlinearity of the individual codon class solutions. Isoleucine, which has an odd number of codons, is the sole exception, the nonlinear ATC codon usage 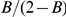 somewhat lower than 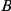 (Figure 5B). This property vindicates the use of 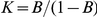 in our model to associate the mutational rate 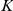 with the overall GC bias as estimate by GC3.

**Figure 7:**
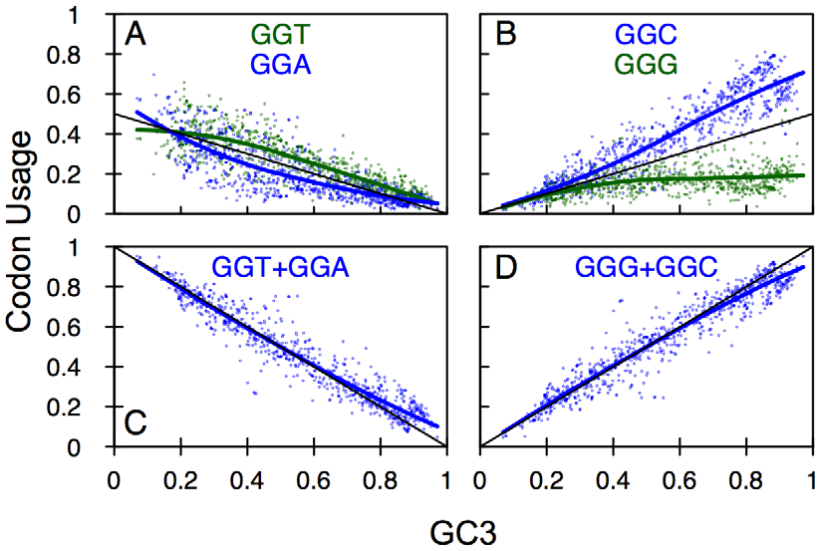
Codon usage frequency for glycine based on prokaryotic data. The x-axis is genomic GC3 as an estimate of GC bias, the y-axis is usage frequency. The colored points and lines correspond to the observed codon usage frequencies for a given GC3 and the corresponding loess fit respectively. The black line is the model prediction. (A) Glycine A/T-ending codon usage. (B) Glycine G/C-ending codon usage. (C) Glycine A/T-ending codon usage summed. (D) Glycine G/C-ending codon usage summed.

Some deviations from the model may be indicative of biologically significant effects; for example GGC and GGG (glycine) (Figure 2) show usage frequencies well above and below that of the model respectively in human, prokaryotic and plant data (see Figure 7 for prokyariotic results, Figures S4 and S5 for human and plant data). The low usage of GGG has been previously noted in Drosophila and attributed to avoidance of guanine runs due to their tendency to form stable mRNA structures which may impede translation [15]. It appears from our results that this effect may extend to bacterial, human and plant codon usage as well.

Many other known influences on codon bias have not been included in our model. Selection for increased transcriptional efficiency can be particularly strong in prokaryotes [6] affects codon usage, and can vary between species. GC skew which is present in the human genome [16], vertebrate mitochondria in general [17], prokaryotes [18] and plants [19] can effect codon bias. Context-specific mutations, where mutations are correlated with neighboring bases [20] may play a role in some of the observed deviations. Nearly-neutral non-synonymous mutations may also play a role: it has been shown that first and second position base content can vary with third position base content in metazoan mitochondria [21]. In addition, even if the model describes equilibrium usage correctly, coding sequences may not all be at or near the equilibrium state described by the model; genes having recently been subject to lateral gene transfer may not yet be in equilibrium with the host genome [22], and recently duplicated genes may not yet be in equilibrium with the GC bias of their host isochore. It may be possible to incorporate some of these effects into future refinements of our model: in particular GC skew effects should be well suited to modeling using our method, however its position dependency poses challenges.

Despite the simplicity of this model and lack of fitted parameters, it broadly captures codon usage trends across multiple branches of life and for a wide range of GC bias. It accounts for 72% of the variation in codon usage in prokaryotes, 64% in plants and 52% in humans and predicts the decrease in usage of AGG and TTG with increasing codon bias. When individual codons are summed together for a class of solutions (0×GC, 1×GC and 2×GC for arginine and leucine (Figures 6D,E,J,K) and G/C- and A/T-ending for the other amino acids (Figures 4E,F, 5C, 7C,D)) our model accounts for 87% of variability in class usage across prokaryotic species, 80% across plant species and 68% across human genes. Thus the influence of GC bias on codon usage appears to be felt most strongly at the level of codon classes rather than the level of individual codons. This effect is puzzling, and it is not clear why it is the case.

Our model establishes that GC bias is the dominant factor in determining codon bias across a broad variety of life and that the form of the influence admits a particularly simple explanation. This model provides a natural null model for codon bias subject to GC mutational bias, relative to which further studies of codon usage may be measured.

## Materials and Methods

Codon usage frequencies for prokaryotic and plant species were downloaded from the CUTG database [23] based on the NCBI GenBank Flat File Release 160.0 [24]. The CUTG gbbct.spsum bacterial data set containing both bacterial and archaean species codon data and the gbpln.spsum plant codon usage data were used. These records sum the codon usage for all nuclear coding sequences in GenBank per species. Though species records can contain duplicated genes, they provide a reasonable estimate of the genomic codon usage for each species, including those for which full, annotated genomic sequences are unavailable. For the 196 plant species and 897 prokaryotic species using the standard genetic code (NCBI genetic code 11) whose entries were based on 50 or more coding sequences (CDS), codon usage frequency per amino acid was calculated.

v54_36p of the Ensembl [25] human genome database was used as the source of genomic sequences accessed via the Ensembl Perl API [26]. For each amino acid, the longest CDS for each of the 20884 protein-coding genes annotated in Ensembl as known containing at least ten codons for that amino acid were chosen for the respective analyses.

For graphs using LOESS (locally estimated scatter plot smoothing) fits, the R 2.10.0 [27] loess function was used to generate the fit to the data (colored lines on graphs) with default parameters (span = 1.0, degree = 2, least-squares fitting).

Percent variance from the model was calculated on a per-codon or per model-class basis by calculating one minus the variance of the difference between the model and the observations divided by the variance of the observations. Averages were then generated for all codons and model classes, each weighted by the number of codons used in the calculation.

The Harvey-Collier test for functional misspecification [28] as implemented in the R lmtest package [29] was used to determine the degree of nonlinearity of codon usage as a function of GC3.

## Supporting Information

Figure S1 Human per-amino acid GC3 vs. GC3 graphs.
(2.00 MB PDF)

Figure S2 Plant per-amino acid GC3 vs. GC3 graphs.
(0.10 MB PDF)

Figure S3 Prokaryote per-amino acid GC3 vs. GC3 graphs.
(0.22 MB PDF)

Figure S4 Human per-amino acid codon frequency vs. GC3 graphs.
(6.69 MB PDF)

Figure S5 Plant per-amino acid codon frequency vs. GC3 graphs.
(0.27 MB PDF)

Figure S6 Prokaryote per-amino acid codon frequency vs. GC3 graphs.
(0.64 MB PDF)

Table S1 Tables of codon usage variance from model for human genes, plant and prokaryote genomes.
(0.08 MB XLS)
