# Supplementary material for: A General Model of Codon Bias Due to GC Mutational Bias
Source: PLoS One. 2010 Oct 27;5(10):e13431. doi: 10.1371/journal.pone.0013431 (PMC2965080; doi:10.1371/journal.pone.0013431)
Supplement: Figure S1 — Human per-amino acid GC3 vs. GC3 graphs. (2.00 MB PDF) [file pone.0013431.s001.pdf]

Human per-amino acid GC3 vs GC3 graphs

Supplemental data for the publication Palidwor et al, 2010

This file contains graphs of per-amino acid GC3 vs GC3 for human protein coding genes based on Ensembl v54_36p.

The x-axis of each graph is GC3, the y-axis is protein GC3 (per-amino acid). Each point represents a single protein coding gene.

The blue line is the model prediction, the red line a loess fit to the observed data.

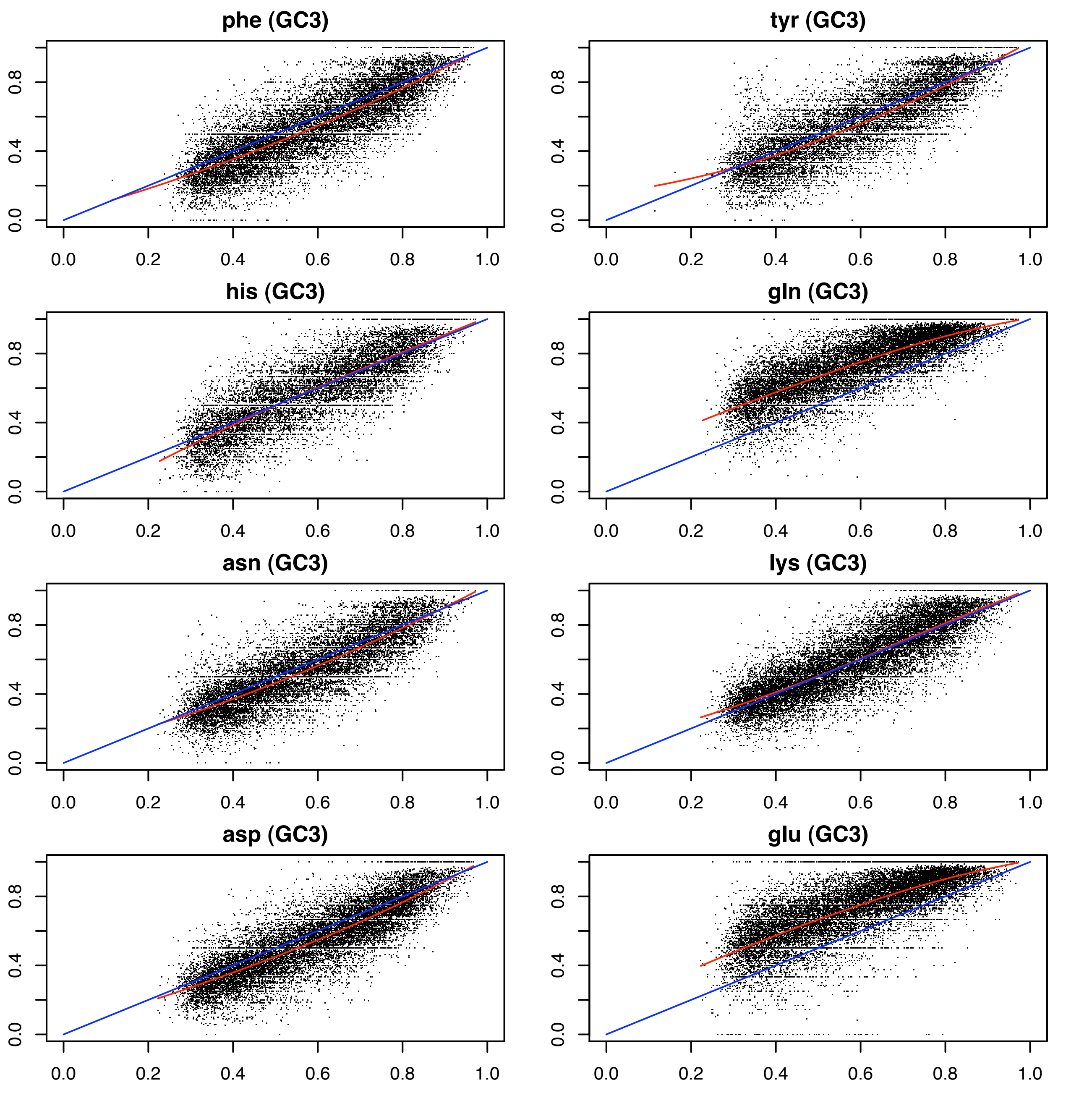

phe (GC3)
tyr (GC3)
his (GC3)
gln (GC3)
asn (GC3)
lys (GC3)
asp (GC3)
glu (GC3)
0.0
0.4
0.8
0.0
0.2
0.4
0.6
0.8
1.0

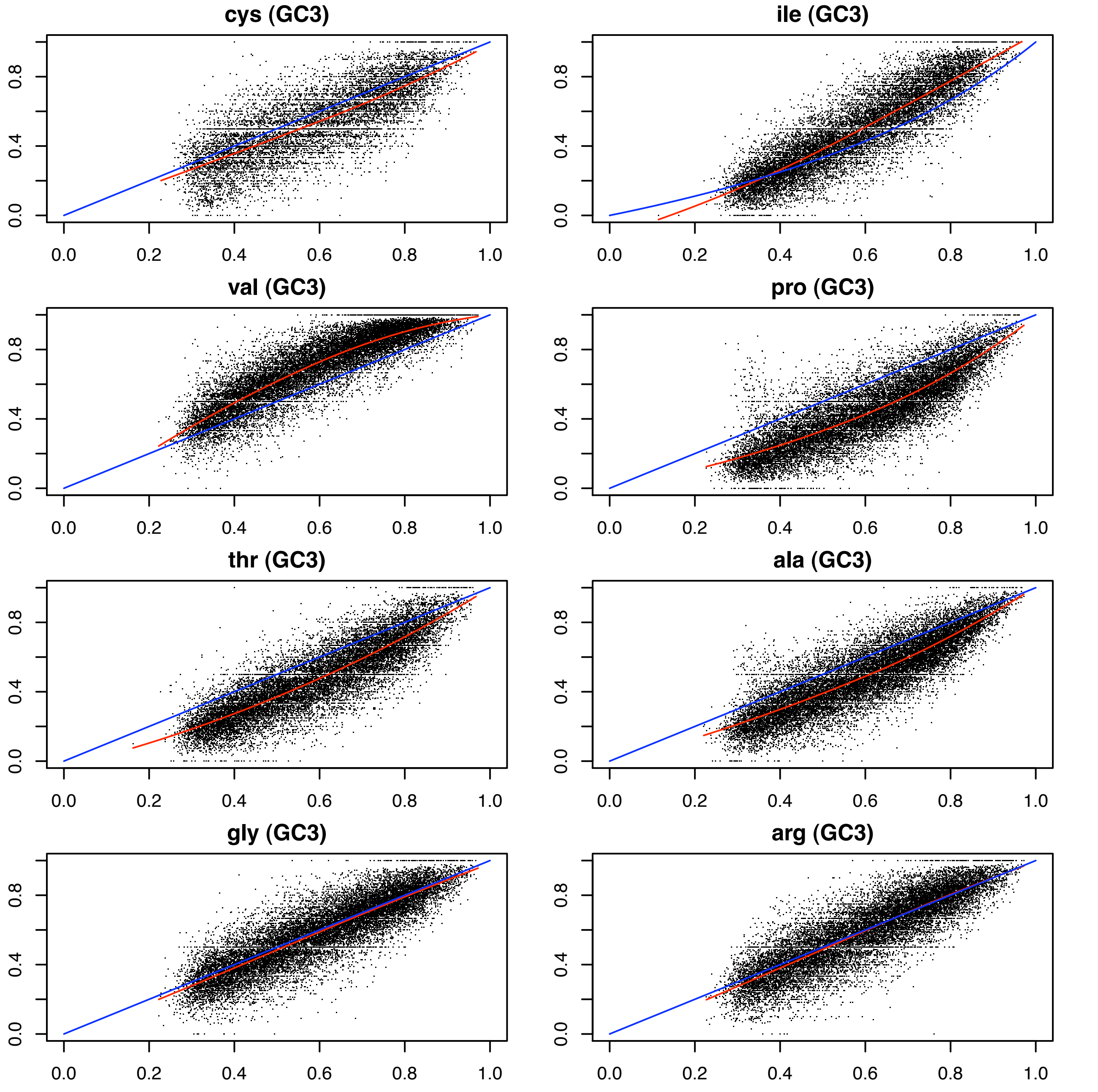
cys (GC3)
ile (GC3)
val (GC3)
pro (GC3)
thr (GC3)
ala (GC3)
gly (GC3)
arg (GC3)
0.0
0.2
0.4
0.6
0.8
1.0

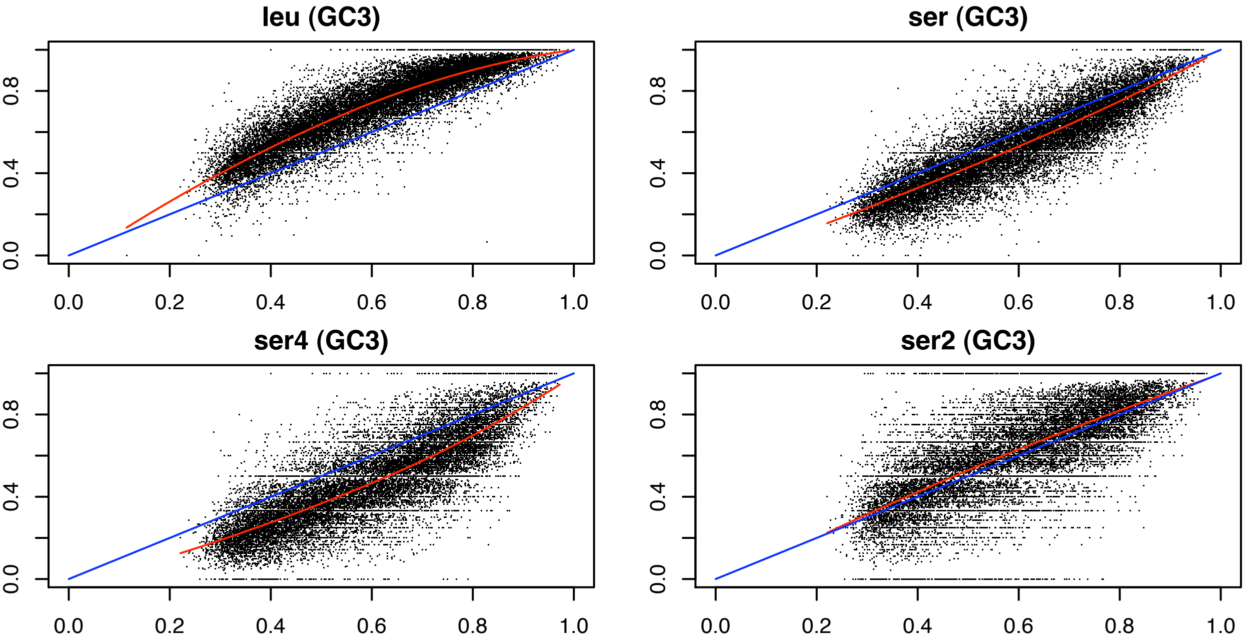

leu (GC3)
ser (GC3)
ser4 (GC3)
ser2 (GC3)
0.0
0.4
0.8
0.0
0.2
0.4
0.6
0.8
1.0
