# Supplementary material for: A General Model of Codon Bias Due to GC Mutational Bias
Source: PLoS One. 2010 Oct 27;5(10):e13431. doi: 10.1371/journal.pone.0013431 (PMC2965080; doi:10.1371/journal.pone.0013431)
Supplement: Figure S3 — Prokaryote per-amino acid GC3 vs. GC3 graphs. (0.22 MB PDF) [file pone.0013431.s003.pdf]

Prokaryotic per-amino acid GC3 vs GC3 graphs

Supplemental data for the publication Palidwor et al, 2010

The x-axis of each graph is GC3, the y-axis is codon frequency (per-amino acid) for prokaryotic genomes based on the gbbct.spsum CUTG codon usage data derived from the NCBI GenBank Flat File Release 160.0.

The x-axis of each graph is GC3, the y-axis is codon frequency (per-amino acid). Each point represents a single prokaryotic genome.

The blue line is the model prediction, the red line a loess fit to the observed data.

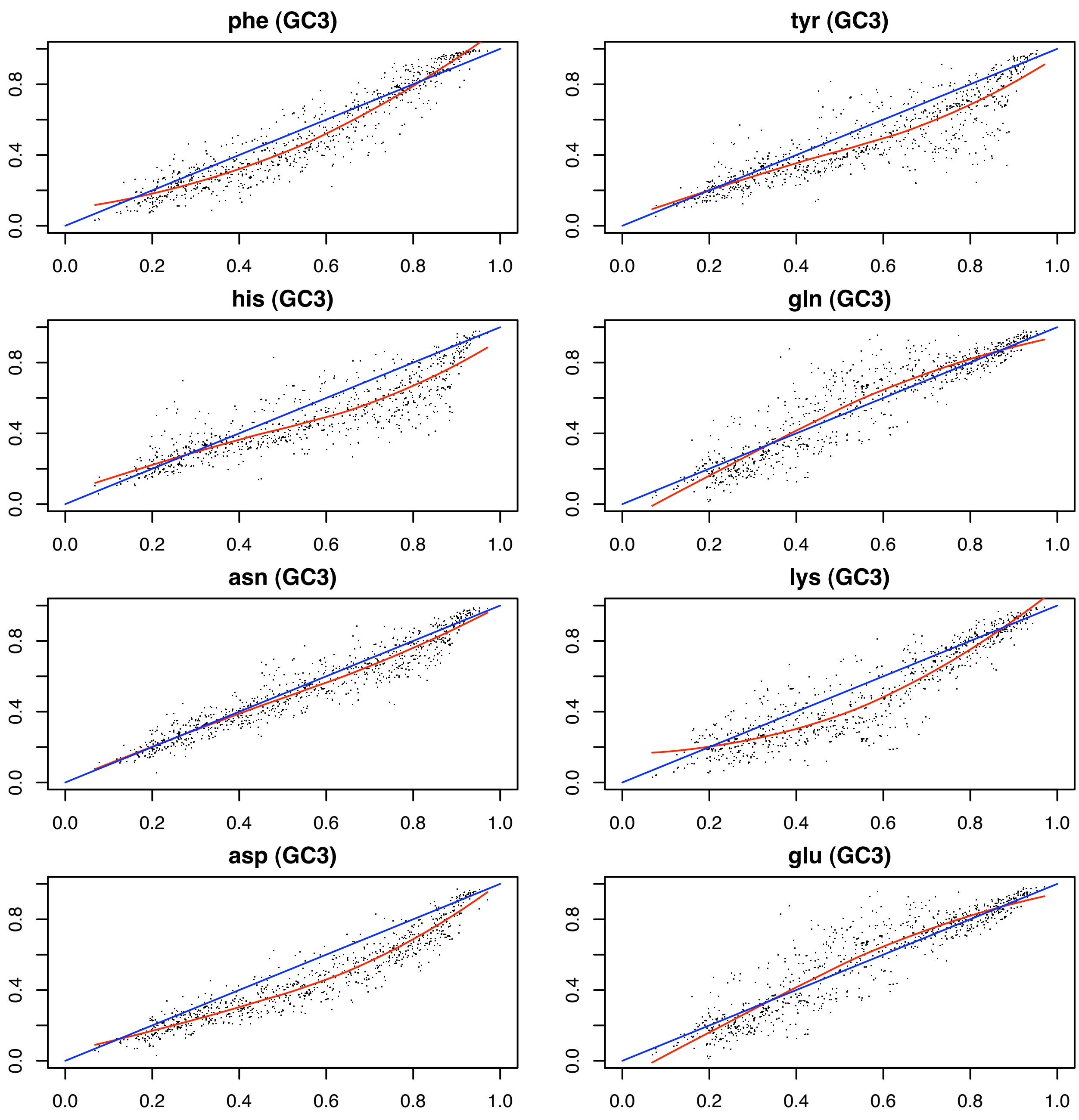

phe (GC3)
tyr (GC3)
his (GC3)
gln (GC3)
asn (GC3)
lys (GC3)
asp (GC3)
glu (GC3)
0.0
0.2
0.4
0.6
0.8
1.0

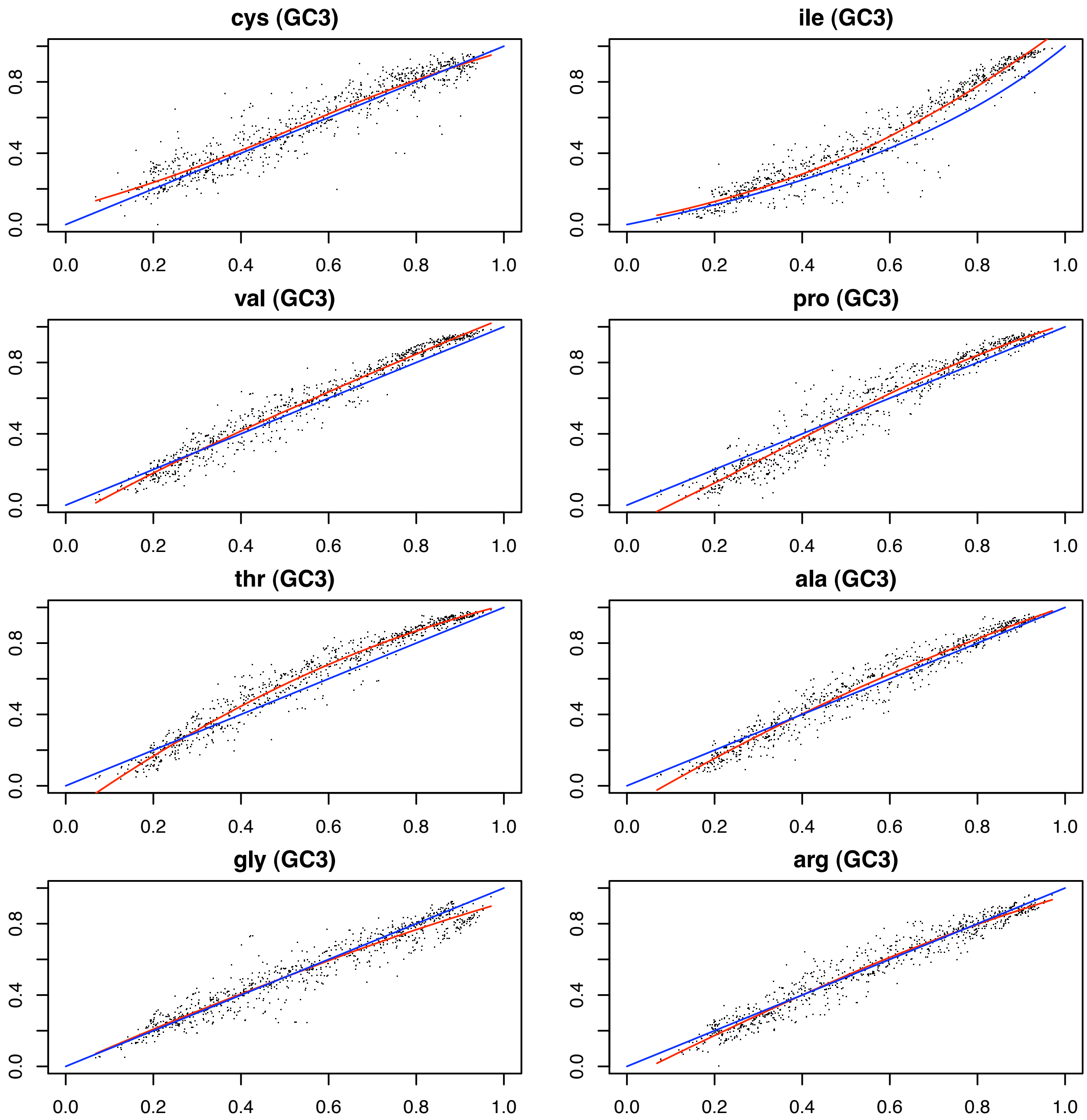
cys (GC3)
ile (GC3)
val (GC3)
pro (GC3)
thr (GC3)
ala (GC3)
gly (GC3)
arg (GC3)
0.0
0.4
0.8
0.0
0.2
0.4
0.6
0.8
1.0

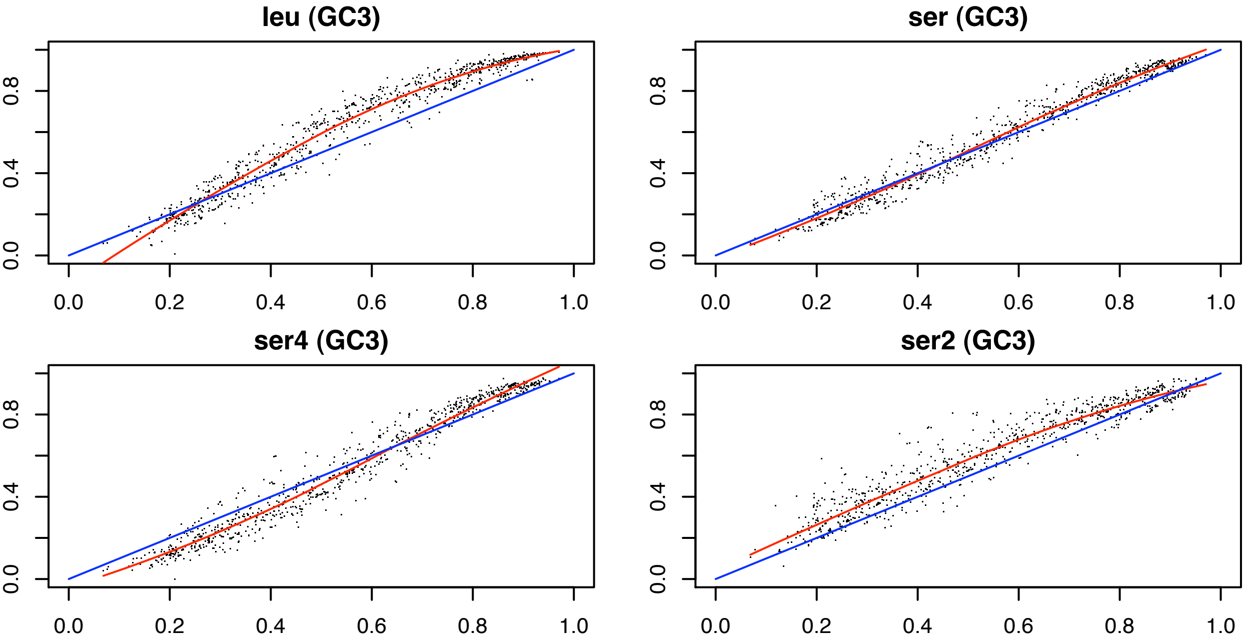
leu (GC3)
ser (GC3)
ser4 (GC3)
ser2 (GC3)
0.0
0.4
0.8
0.0
0.2
0.4
0.6
0.8
1.0
