# Supplementary material for: A General Model of Codon Bias Due to GC Mutational Bias
Source: PLoS One. 2010 Oct 27;5(10):e13431. doi: 10.1371/journal.pone.0013431 (PMC2965080; doi:10.1371/journal.pone.0013431)

Prokaryotic per-amino acid codon frequency vs GC3 graphs

Supplemental data for the publication Palidwor et al, 2010

This file contains graphs of codon frequency vs GC3 for prokaryotic genomes based on the gbbct.spsum CUTG codon usage data derived from the NCBI GenBank Flat File Release 160.0.

The x-axis of each graph is GC3, the y-axis is codon frequency (per-amino acid). Each point represents a single prokaryotic genome.

The blue line is the model prediction, the red line a loess fit to the observed data.

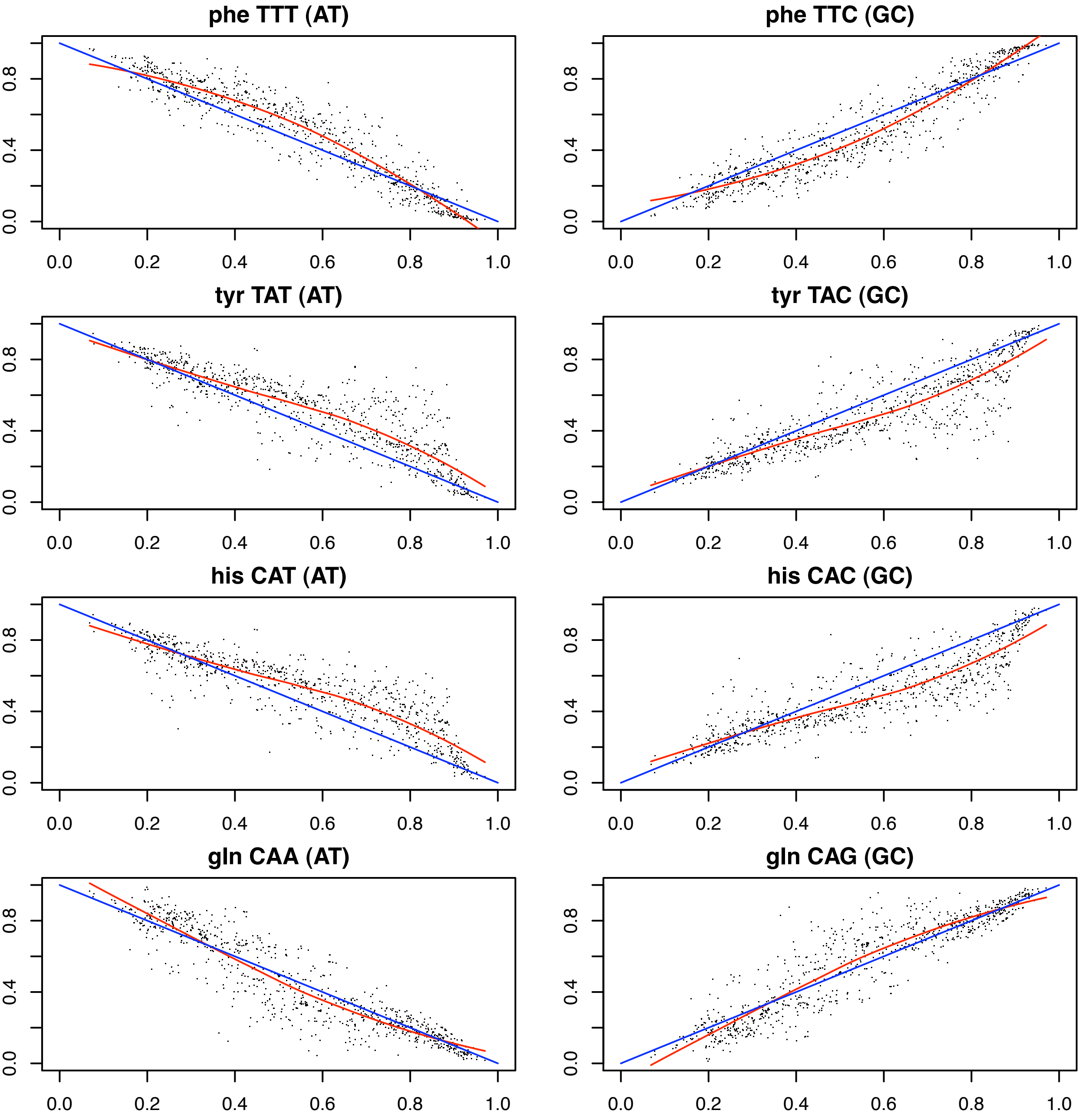
phe TTT (AT)
phe TTC (GC)
tyr TAT (AT)
tyr TAC (GC)
his CAT (AT)
his CAC (GC)
gln CAA (AT)
gln CAG (GC)
0.0
0.2
0.4
0.6
0.8
1.0

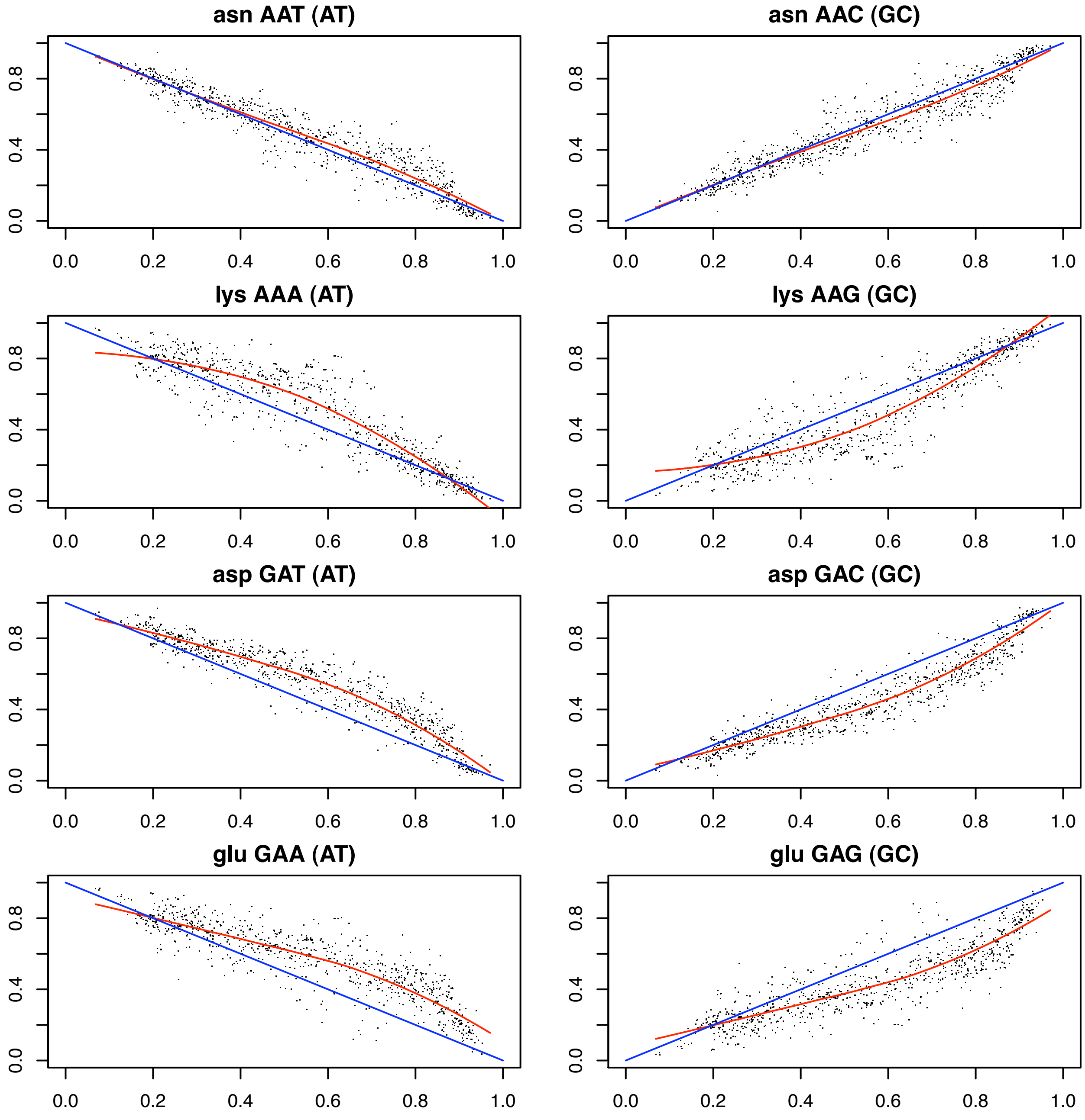
asn AAT (AT)
asn AAC (GC)
lys AAA (AT)
lys AAG (GC)
asp GAT (AT)
asp GAC (GC)
glu GAA (AT)
glu GAG (GC)
0.0
0.4
0.8
0.0
0.2
0.4
0.6
0.8
1.0

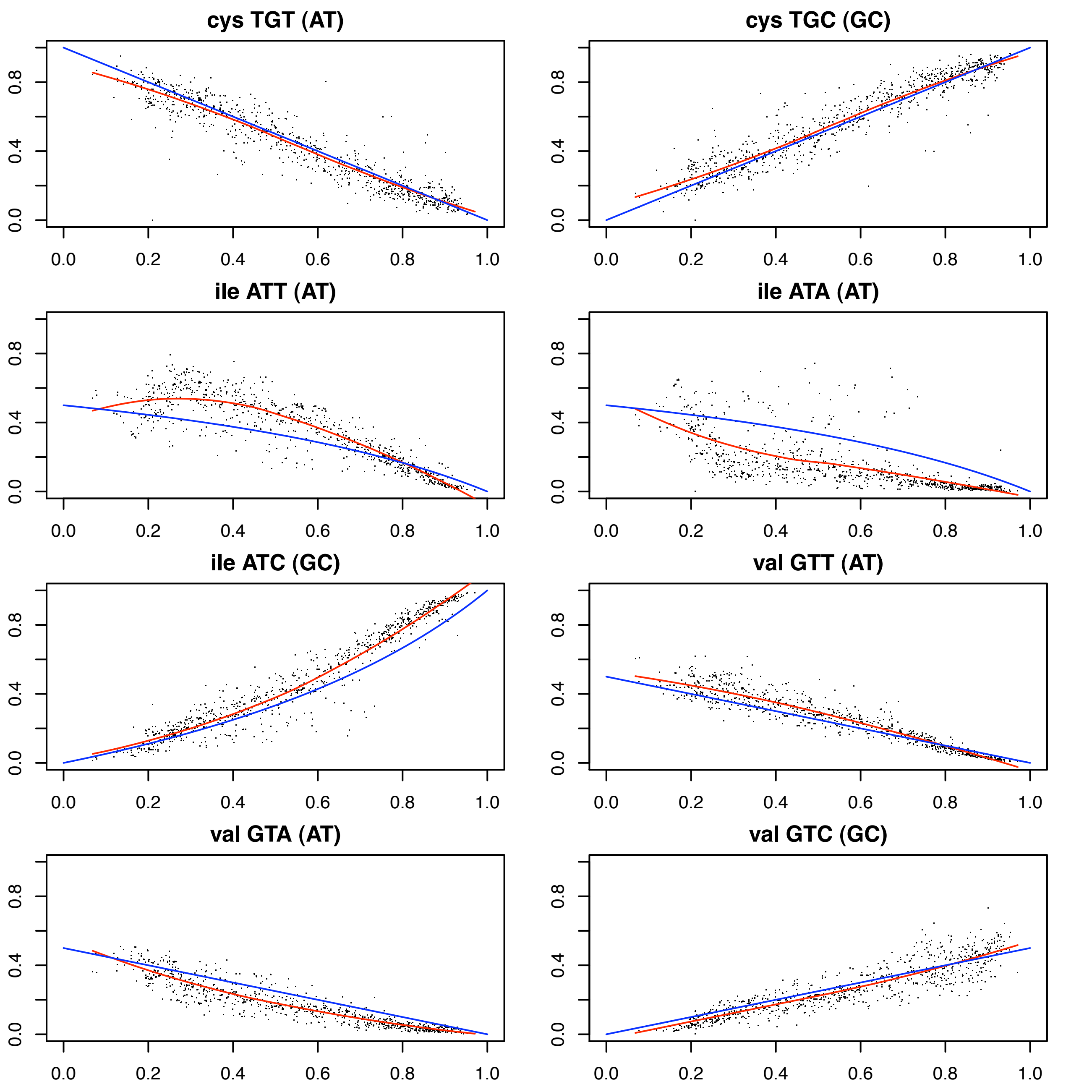
cys TGT (AT)
cys TGC (GC)
ile ATT (AT)
ile ATA (AT)
ile ATC (GC)
val GTT (AT)
val GTA (AT)
val GTC (GC)
0.0
0.4
0.8
0.0
0.2
0.4
0.6
0.8
1.0

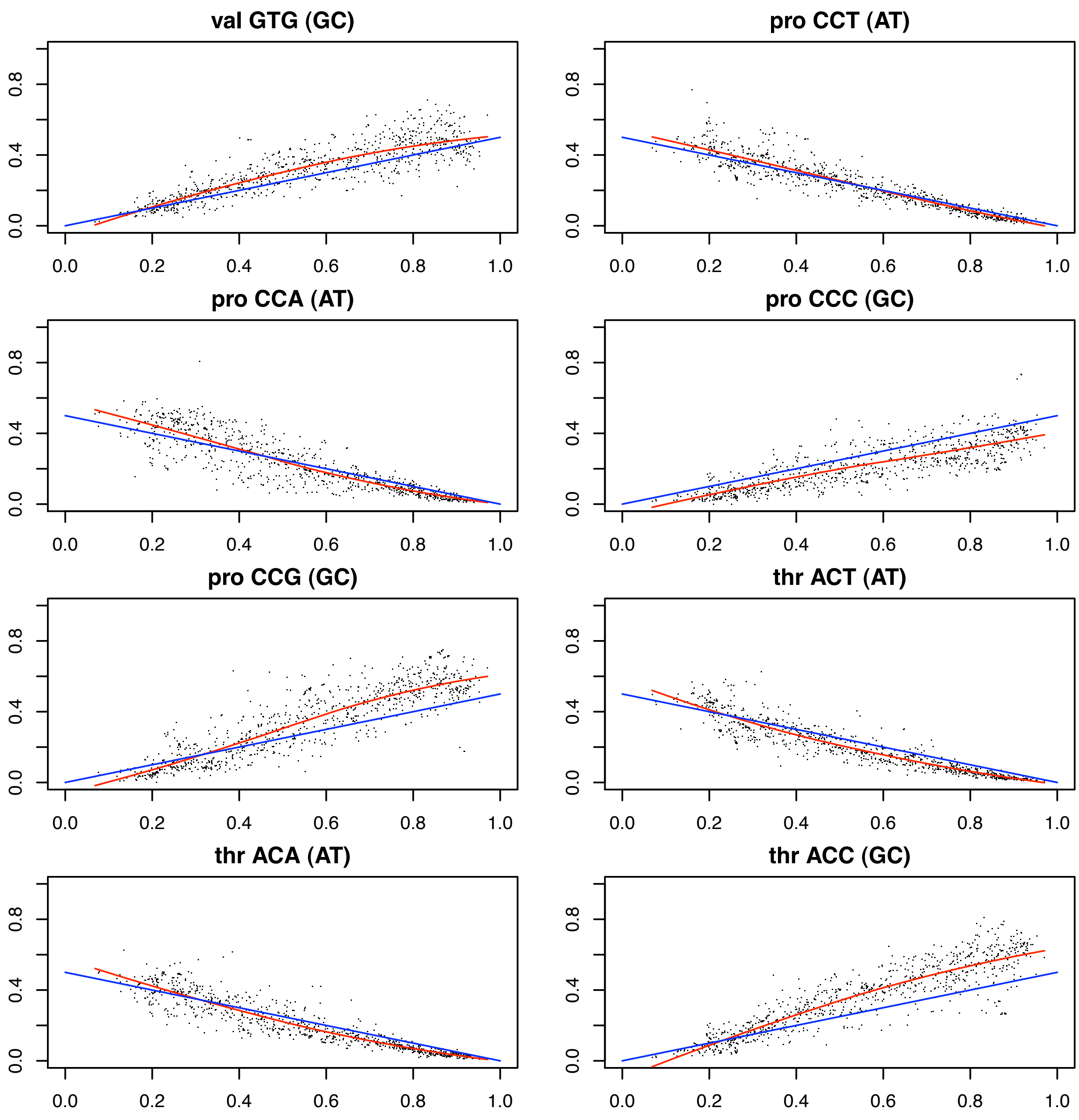
val GTG (GC)
pro CCT (AT)
pro CCA (AT)
pro CCC (GC)
pro CCG (GC)
thr ACT (AT)
thr ACA (AT)
thr ACC (GC)
0.0
0.4
0.8
0.0
0.2
0.4
0.6
0.8
1.0

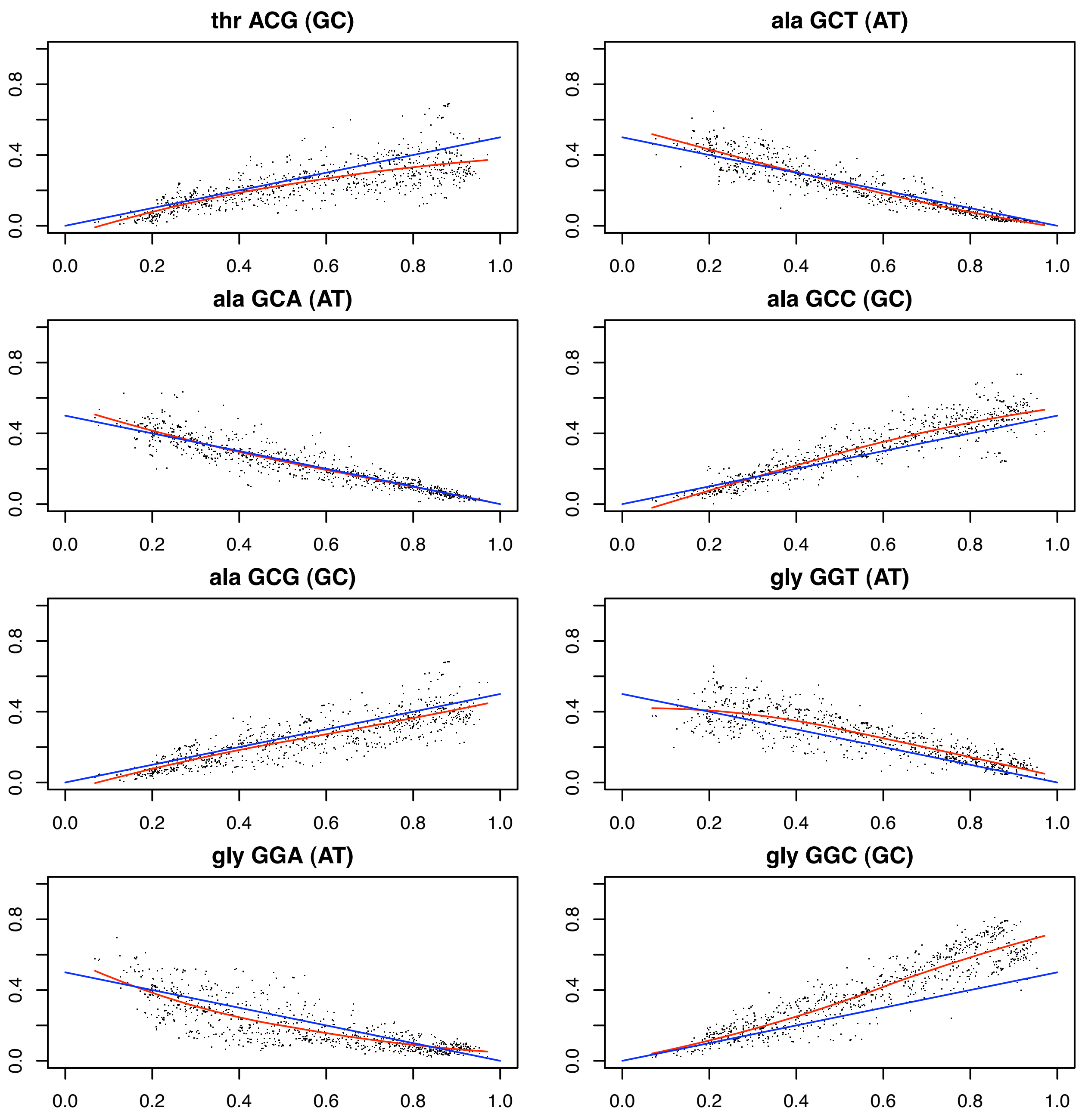
thr ACG (GC)
ala GCT (AT)
ala GCA (AT)
ala GCC (GC)
ala GCG (GC)
gly GGT (AT)
gly GGA (AT)
gly GGC (GC)
0.0
0.4
0.8
0.0
0.2
0.4
0.6
0.8
1.0

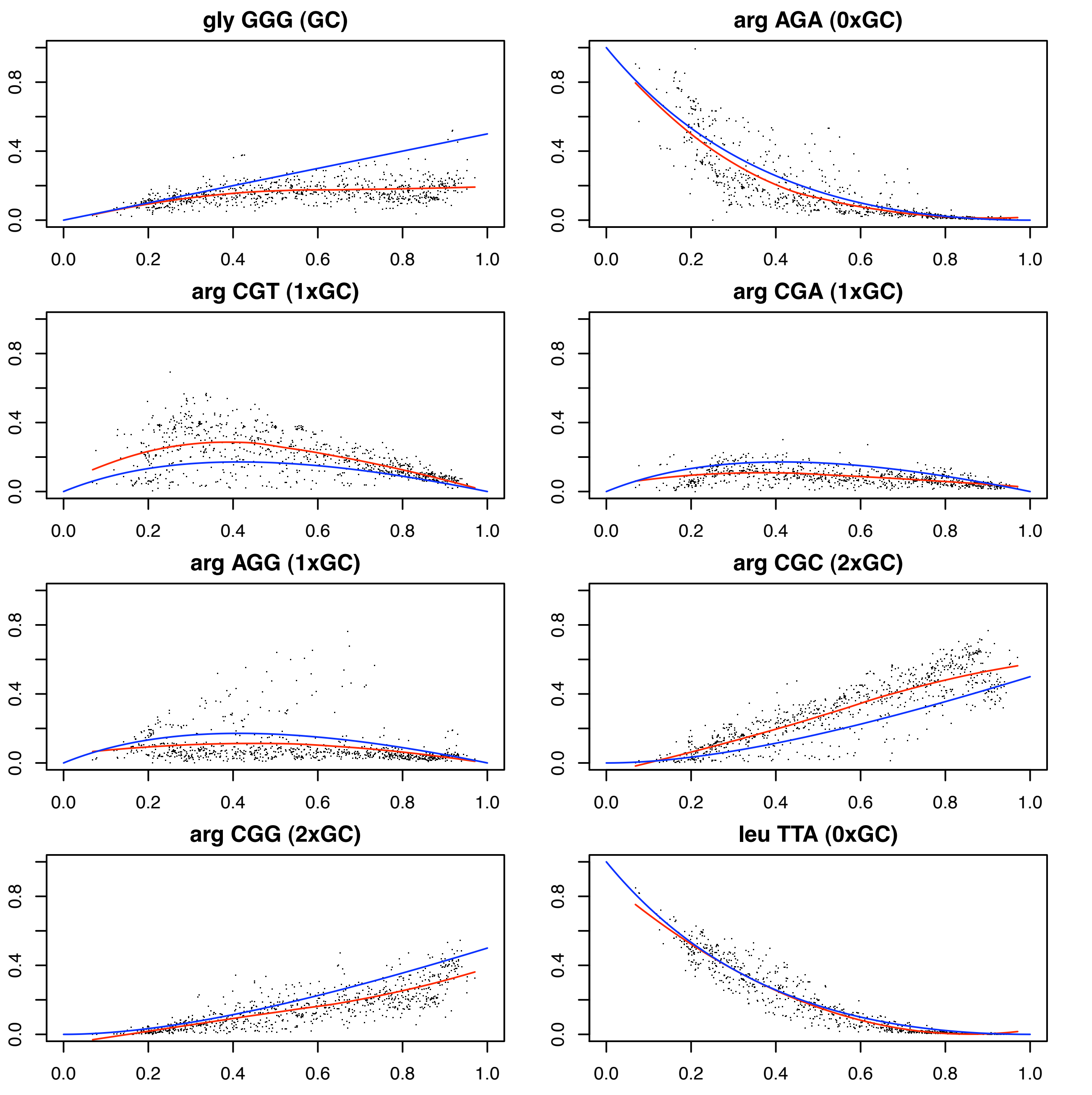
gly GGG (GC)
arg AGA (0xGC)
arg CGT (1xGC)
arg CGA (1xGC)
arg AGG (1xGC)
arg CGC (2xGC)
arg CGG (2xGC)
leu TTA (0xGC)
0.0
0.4
0.8
0.0
0.2
0.4
0.6
0.8
1.0

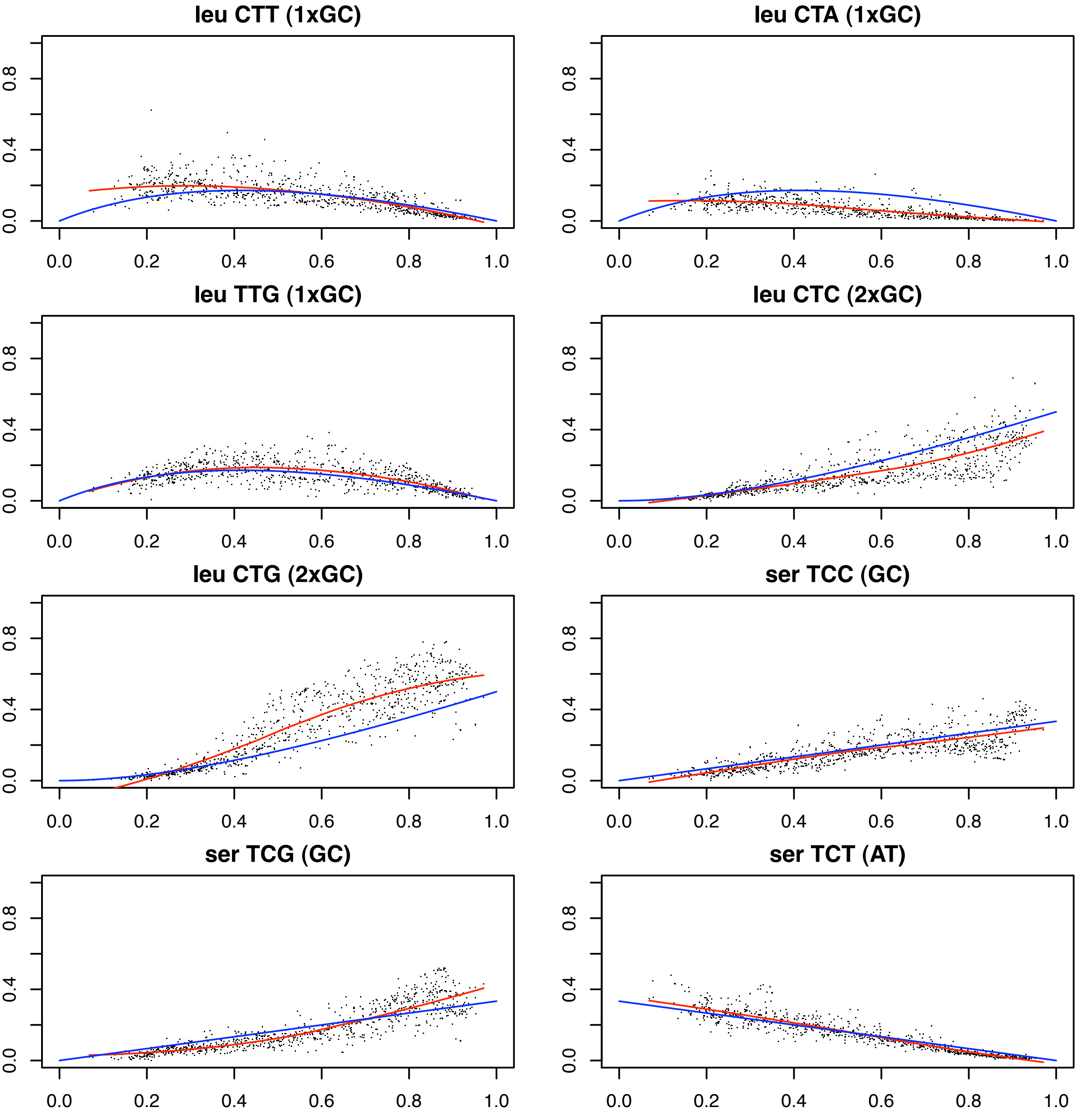
leu CTT (1xGC)
leu CTA (1xGC)
leu TTG (1xGC)
leu CTC (2xGC)
leu CTG (2xGC)
ser TCC (GC)
ser TCG (GC)
ser TCT (AT)
0.0
0.4
0.8
0.0
0.2
0.4
0.6
0.8
1.0

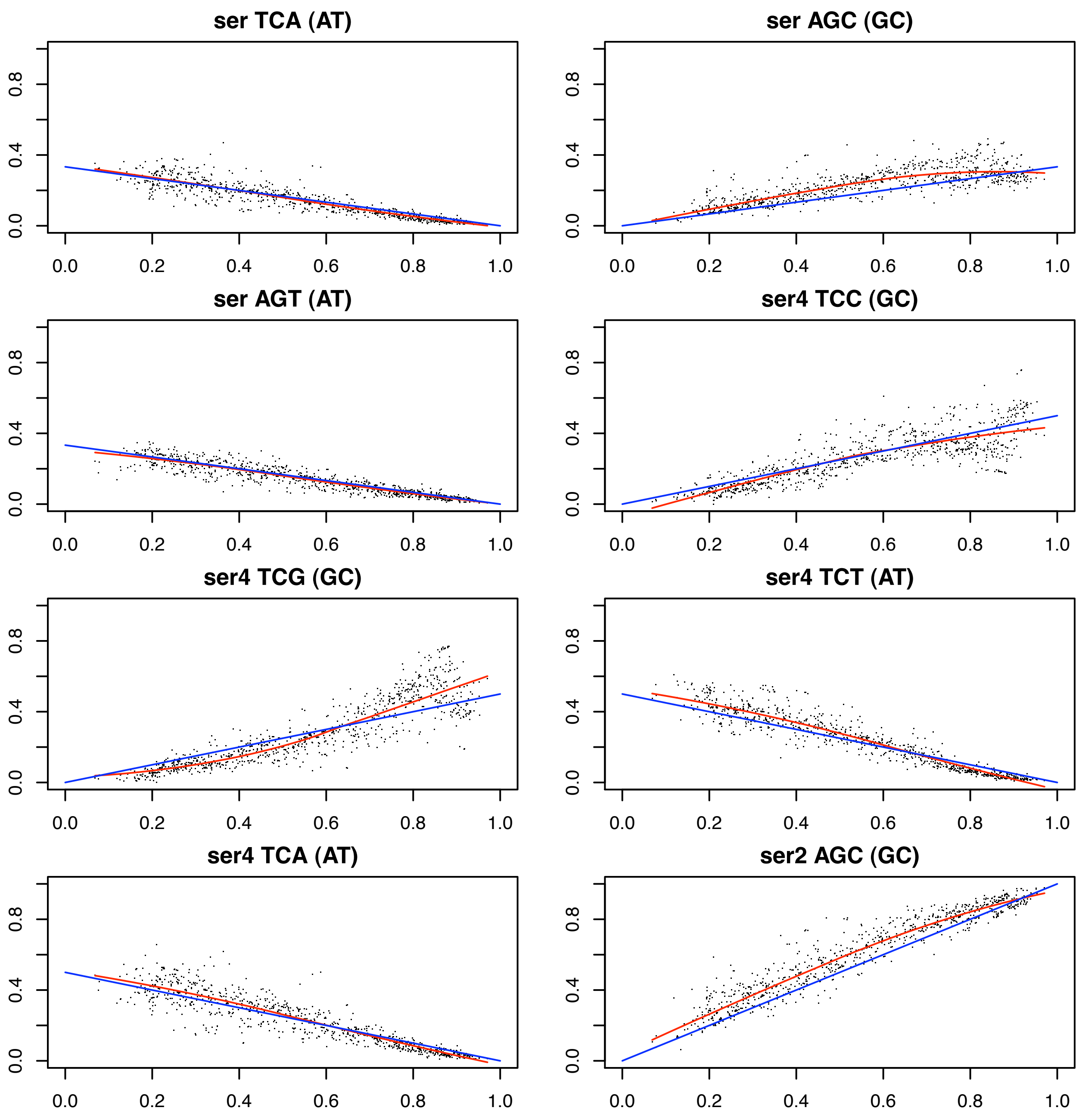

ser TCA (AT)
ser AGC (GC)
ser AGT (AT)
ser4 TCC (GC)
ser4 TCG (GC)
ser4 TCT (AT)
ser4 TCA (AT)
ser2 AGC (GC)
0.0
0.4
0.8
0.2
0.6
1.0

ser2 AGT (AT)

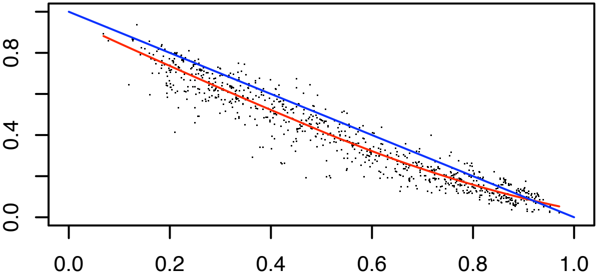

Supplement: Figure S6 — Prokaryote per-amino acid codon frequency vs. GC3 graphs. (0.64 MB PDF) [file pone.0013431.s006.pdf]
